# Supplementary material for: Complexome profiling of the Chlamydomonas psb28 mutant reveals TEF5 as an early PSII assembly factor
Source: Plant Cell. 2025 Mar 17;37(6):koaf055. doi: 10.1093/plcell/koaf055 (PMC12164590; doi:10.1093/plcell/koaf055)
Supplement: koaf055_Supplementary_Data [file koaf055_supplementary_data.zip › psb28_interactive.zip!/plots/intensity/qualityPlots/peptides/_Cre12.g484700.t1.2.html]

### Cre12.g484700.t1.2

Trivial name:   
SeqCoverage [median]: 70.30  
Mass: 13.77 kDa  
  
 

| Sequence | MissedCleavage | Mass [Da] | wtIntensity | muIntensity | Score |
| --- | --- | --- | --- | --- | --- |
| AEALKALEK | 1 | 971.57 | 6541.18 | 21597.08 | 153.05 |
| AVVPHDSPK | 0 | 948.50 | 309.63 | 878.17 | 77.67 |
| FSPGTQPVK | 0 | 959.51 | 9604.79 | 7153.80 | 87.48 |
| KFSPGTQPVK | 1 | 1087.60 | 1144.77 | 2379.05 | 96.33 |
| LVYDIK | 0 | 749.43 | 1437.48 | 1686.32 | 98.03 |
| LYAGMPTKPEQVK | 1 | 1460.77 | 1832.31 | 0.00 | 55.31 |
| NVTRPLIMPTR | 1 | 1296.73 | 22560.36 | 23735.99 | 96.73 |
| NVTRPLIMPTRGY | 2 | 1516.82 | 2200.11 | 609.63 | 110.92 |
| SIPEFEHYLPMGLEYR | 0 | 1979.95 | 8956.03 | 1187.51 | 52.52 |
| TTFDFDKLYAGMPTKPEQVK | 2 | 2315.15 | 1438.47 | 3053.13 | 85.51 |
|  |  |  | 56025.1 | 62280.7 |  |
